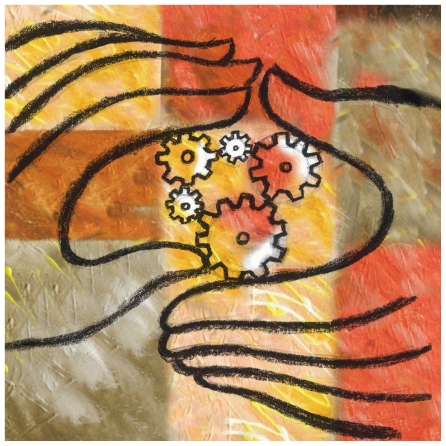# The Perception Factor: Climate Change Gets Personal

**DOI:** 10.1289/ehp.118-a484

**Published:** 2010-11

**Authors:** Catherine M. Cooney

**Affiliations:** **Catherine M. Cooney** is a science writer living in Washington, DC

Summer 2010 saw a new suite of climate change studies from the National Academy of Sciences (NAS) with the stark conclusion that “Climate change is occurring, is caused largely by human activities, and poses significant risks for—and in many cases is already affecting—a broad range of human and natural systems.”^1^ The NAS series received a boost from separate research indicating that up to 98% of the climate researchers most actively publishing agree with the tenets of anthropogenic climate change outlined by the Intergovernmental Panel on Climate Change.^2^ At about the same time, however, Senator Harry Reid (D–NV) announced he couldn’t find the votes to pass legislation designed to reduce greenhouse gas emissions from major sources.^3^ Meanwhile, in California, a fiercely debated jobs bill called Proposition 23 seeks to suspend—some say effectively repeal—the state’s ambitious greenhouse gas legislation until unemployment drops to no more than 5.5% for a full year.^4^

This whopping disconnect between legislators and the scientific community could be a signal that it is time for a new path toward climate change mitigation and adaptation that more directly involves the public. Many researchers interested in global warming are wondering: just what might it take to encourage individuals in the United States to think more seriously about climate change?

## A New Era for Climate Change Science

To find out, the NAS recommended tapping into social science, which can be used to describe people’s perceptions of critical facts and their goals when making choices.^5^ This research has been under way for decades and saw a relatively small but significant boost in the 1970s during the energy crisis, says Paul Stern, director of the National Research Council Committee on the Human Dimension of Global Climate Change. The focus then was on energy consumption and conservation in households, but funding dried up quickly after oil prices went down, Stern says.

Recently there has been a surge in published social science papers looking into the U.S. public’s perception of climate change, notes Edward Maibach, director of the Center for Climate Change Communication at George Mason University. Maibach says climate researchers are eager for social scientists to tell them what they know about human behavior and why a majority of Americans are not taking action that could significantly reduce their own carbon footprint.

Renewed interest in social science was expressed clearly by the NAS reports issued this summer.^1^ These reports, known as the America’s Climate Choices (ACC) series, summarize the science on global warming and recommend that the country focus its efforts on areas where the most severe impacts are expected to occur.^6^ They also discuss current U.S. attitudes toward climate change and suggest communication should improve between scientists and the general public about global warming science and its impacts. The report *Advancing the Science of Climate Change* urges that climate change science incorporate disciplinary and interdisciplinary research across the physical, social, biological, health, and engineering sciences.^1^ This integrated approach should help put the science into perspective not just for a struggling public but also for policymakers ranging from city planners to members of Congress.

Pamela Matson, dean of the School of Earth Sciences at Stanford University and chairwoman of the *Advancing the Science of Climate Change* study panel, says her group concluded there already exists a significant amount of research on understanding climate change and its impacts, both of which are critical for a national climate change program. But Matson says we also need to try to complement existing research with data on new or understudied elements of climate change science—areas such as risk communication to help people better understand climate change and behavioral science to improve understanding of individual, societal, and institutional factors that shape decisionmaking. “I would say this is the most significant recommendation that we make,” she says.

Thomas Dietz, assistant vice president for environmental research at Michigan State University, who cochaired the *Advancing the Science of Climate Change* panel, agrees. “There is still a tremendous amount of work that has to be done on the physical sciences,” Dietz says. But it’s also important for researchers to confer and collaborate with others outside their discipline, he explains. “We really need that type of integrated scientific approach, not the old silos that we are used to dealing with.”

The number of peer-reviewed studies on human behavior regarding energy use and climate change is relatively small, notes Baruch Fischhoff, a professor of social and decision sciences at Carnegie Mellon University. “I think we’ve had a major institutional failure to serve and understand the public, then have blamed the public for our failure to communicate effectively,” Fischhoff says. “When you think about how big the problems are and how much money we spend on other aspects of them, it is kind of laughable. We are largely flying blind when it comes to understanding essential human behaviors.”

## “Six Americas” and Other Perceptions

Researchers do understand some of why people make the decisions they do, and they agree on several overarching themes when it comes to climate change: Americans often are very selective about which sources of information they trust; they have incomplete, often oversimplified information; they don’t believe individual actions will make a difference; and/or they believe climate change won’t ever affect them or the people they know. But like all things dealing with climate change, human decisionmaking in this area is complex.

Sammy Zahran, codirector of the Center for Disaster and Risk Analysis at Colorado State University, says the reaction of Americans who don’t support efforts to control global warming isn’t so surprising. In 2008, he tried to get at what it is that encourages local officials to reduce carbon emissions even though they might believe the expected net benefits for their city are low.^7^ His team found that cities in regions that are the most vulnerable to the impacts of climate change but that emit the least greenhouse gases (such as Providence, Rhode Island) are most likely to act to reduce their carbon emissions. On the other hand, cities in less vulnerable areas with sources that emit high levels of greenhouse gases (such as Jefferson County, Arkansas, home to the coal-fired White Bluff Power Plant) are significantly less likely to agree to take mitigation action. Zahran argues that this study shows how difficult it is for the United States as a whole to take action on a problem when individuals in one area perceive their burden to be disproportionate to that of others in the country.

Zahran has also investigated public support of climate change policies. By and large the statistical results behaved as expected: people are more likely to support costly climate change policies if they perceive climate change as threatening their well-being, and they are more likely to support policies if they have confidence in the institutions that regulate climate change outcomes.^8^

National surveys of randomly selected adults paint a picture of how the general public views climate change. A January 2009 poll by the Pew Research Center for the People & the Press discovered that global warming was a low national priority for Americans, ranking dead last behind 20 other concerns such as job loss, terrorism, Medicare, and health care.^9^ One explanation may be that in many people’s minds, climate change has moved away from being a scientific debate to a political one. “This has been exceedingly well established in data . . . that political ideology and some deeply held worldviews related to political identity are currently the biggest factors that determine a person’s view of climate change,” Maibach says.

Another set of surveys digs deeper into the question of why Americans may not believe global warming is a serious concern. In the most recent survey in June 2010, Anthony Leiserowitz, director of the Yale Project on Climate Change, and Maibach polled more than 1,000 adults and found what they describe as “global warming’s Six Americas.”^10^ These six distinct responses to climate change range from those who are engaged with the issue and who take steps to reduce their emissions (the Alarmed) to those who actively deny the reality of climate change and don’t think any action is needed (the Dismissive). The survey showed that 13% of respondents were Alarmed and eager to take action, 28% were Concerned, 24% Cautious, 10% Disengaged, 12% Doubtful, and 12% Dismissive.^10^

Within the Alarmed sector, most said they believe global warming is happening and is caused by humans; the Dismissive group either denied it is happening, said the science was not yet proven, or said it was happening but is part of a natural cycle. Yet those in the Dismissive camp are just as likely to engage in energy conservation actions at home (such as turning off lights) as any of the other segments, the survey found. Those in the Dismissive segment are also more likely to say that news accounts of events that cast doubt on climate change science have an impact on their thinking about global warming and support their belief that it is not yet proven.^10^

The survey further reveals there is room for change: Large numbers of those polled, including 65% of those in the Cautious category and and 73% of those in the Disengaged segment, said they could easily change their minds about global warming. Nearly half of all participants—especially the Concerned (57%), Cautious (50%), and Disengaged (63%)—said they need “some more” or “a lot more” information to form a firm opinion about global warming.^10^

## A Formula for Success?

The United States is second only to China in both energy use and carbon emissions, its factories, cars, homes, and power plants contributing approximately 19% of the world’s carbon dioxide emissions.^11^ Perhaps surprisingly, individual homes represent a huge and still largely untapped source of U.S. emissions reductions.^12^ In 2007 Michael Vandenbergh, director of the Climate Change Research Network at Vanderbilt University, found that individual households in the United States contribute roughly a one-third share of total U.S. carbon emissions, accounting for approximately 8% of the world’s total and equaling more than the total emissions of any other country except China, and more than several continents.^13^

Yet homeowners often don’t embrace utility and government-sponsored rebate programs for weatherization, energy-efficient appliances, and solar panels. Stern says a number of financial and nonfinancial barriers affect people’s investments in energy efficiency. For example, people are not likely to install energy efficiency technologies in their homes if it is not easy to do and if they don’t trust the quality of the information. “It is possible to practically give things away for free and have people still not do it,” Stern says.

Vandenbergh, Dietz, Stern, and colleagues have developed what they call a “behavioral wedge theory.”^14^ They took what is understood about behavior and energy use and assessed where policy makers should focus to get the greatest emission reductions and energy use cuts, Vandenbergh says. They concluded that national implementation of proven programs that don’t require new regulations—such as home weatherization and routine vehicle maintenance—eventually could remove from the air an estimated 123 million metric tons of carbon per year. This equals 7.4% of U.S. national emissions or France’s entire carbon output.^15^

Successful policies aimed at homeowners should share several traits, the team found. The information must be accessible and must come from a source deemed trustworthy; the activity required to reduce emissions must be relatively easy to undertake; and the activity also should provide a fairly quick financial payback so the individual is expeditiously rewarded, Stern says.

One way to encourage Americans to adopt a more serious outlook toward climate change is by having medical professionals link health issues and climate change impacts.^16^ In a 2008 survey conducted by Maibach and colleagues, 60% of local public health directors asked said they are seeing health effects related to global warming, and more than 70% said they thought they would see more in the next decade.^17^ “Public health officials have a really important opportunity to explain to people in their jurisdiction . . . that climate change is not just a problem in the future. It is a current problem that will become more pronounced, and our health will suffer,” Maibach says.

Information about the potential health benefits of specific mitigation-related policy actions appears to be particularly compelling for individuals.^16^ For example, using the car less and walking more can not only help reduce emissions but also aid in a person’s fight against obesity.

Everyone interviewed for this story agrees individual changes to reduce greenhouse gases will solve only part of the problem. “We could try to change people’s climate behavior from now until the cows come home, but even if it was successful it won’t truly solve the problem,” Maibach says. “We are going to need policy changes.”

Others are at work exploring steps the federal government can take to reduce greenhouse gas emissions without Congress approving a new bill.^18^ In a July 2010 report the environmental think tank World Resources Institute described the emissions reductions possible under existing authority combined with emissions cut by actions planned at the state level.^19^ According to the report, these actions, if fully implemented, could result in significant reductions approaching President Obama’s goal of cutting U.S. greenhouse gas emissions by 17% below 2005 levels by 2020.

## Embracing a Positive Future

Several web-based movements that link energy-conscious individuals with others in their community have sprung up in recent years.^20^ These nonprofit groups promote community-based projects such as communal gardens for growing produce and bicycle workshops that offer free bike repairs, bike lane maps, and connections with cyclists who commute. They inspire people to act by promising a positive future in which they have some control. It is exactly this positive outlook, combined with the localization of global warming, that has been missing from the public dialogue on climate change, climate communication experts say.^16^

Carolyne Stayton, executive director of one of these web-based groups, Transition US, notes that one goal of this movement is to engage the community in its own “energy descent plan” complete with steps to wean town residents off fossil fuel use. The schemes immerse the locality in a future tailored to its own needs, she says. “Because Transition is so positive, it does bring people together who are facing denial about climate change. It is a very pleasant way of getting your eyes opened,” she adds.

“One thing we don’t have is a narrow prescription of what is the one right answer for everyone who wants to create a clean energy future and respond to climate change. We are a big tent, and we are encouraging people to take whatever action is appropriate in their community,” says Anna Goldstein, U.S. campaign manager for another online-based group known as 350.org. This group, founded by environmentalist and author Bill McKibben, recently helped rally more than 7,000 events in 188 countries for the 10/10/10 Global Work Party, in which people in locales all over the globe joined in community-based sustainability and clean energy projects on 10 October 2010.^21^

Satisfying an individual’s need for trust is a given with these movements because information routinely comes from other people in the community. But they don’t offer any answers to questions concerning how humans make decisions. “What we do have is a lot of energy and excitement about a future in which community needs are met before a disaster arises,” Stayton says.

Stayton and Goldstein freely admit they are most often working with motivated individuals, not those in the Dismissive camp. But they believe they provide a channel for anyone interested in policy change. Pressuring political leaders is a key element of 350.org, Goldstein says, and she stresses that having all the 10/10/10 activities occur on one day was done to make a splash in the news. This approach will be successful “not because we can stop climate change one bike path at a time, but because we need to make a sharp political point to our leaders: we’re getting to work, what about you?” McKibben noted in his blog.^22^

But Maibach says community-based groups, although “absolutely critical,” are not yet sufficiently successful in getting people to contact policymakers. His research shows people are more apt to choose products as a solution rather than contact their elected representatives.^10^ “Many people don’t think contacting their elected representatives will make a difference. But elected officials and their staff say the opposite,” Maibach says. Tellingly, he adds, “They tell us they get lots of contact from angry voters who are against climate change policy, but very little saying that it is a good idea.”

There are still many things researchers don’t know about human decisionmaking, especially with regard to climate change policy, but they are sure they can take on the challenge. “We need to get beyond the idea that the way to look for emission reductions is in the same place we have already looked,” Vandenbergh says. “We need to look at household emissions and treat them with the same amount of attention and policy resources as any other emitter source.” He says the key to successful individual changes will be government policies that have a national impact but are implemented locally by multiple organizations. “I think it can be done and done very well,” Vandenbergh adds, “but it requires tremendous attention.”

## America’s Climate Choices

The National Academy of Sciences (NAS) released a one-of-a-kind suite of reports on climate change in June 2010.^1^ Called the America’s Climate Choices (ACC) report series, the documents provide crucial information for the members of the U.S. Global Change Research Program, which coordinates and integrates federal research on climate and global changes. “What we see in the ACC series is really an affirmation that the human health impacts are one of the most important impacts for society from climate change,” says John Balbus, senior advisor for public health at the National Institute of Environmental Health Sciences, who represents the U.S. Department of Health and Human Services on the Global Change Research Program.

Each of the first four reports, *Advancing the Science of Climate Change*, *Limiting the Magnitude of Climate Change*, *Adapting to the Impacts of Climate Change*, and *Informing an Effective Response to Climate Change*, was developed by a separate panel of experts. A fifth report, *Informing an Effective Response to Climate Change*, builds on the recommendations of each of the four other reports and provides a scientific framework for shaping climate change policy choices.

Congress requested the series of five reports more than two years ago, and in doing so handed the academy “an unusually broad request—to analyze what we know about climate change, what are the causes, and how should the nation respond,” says NAS president Ralph J. Cicerone. The reports were developed with input from more than 90 experts in academia, business, industry, government, nongovernmental organizations, and the international community working *pro bono*. The overarching study plan was crafted during the America’s Climate Choices Summit held in March 2009.^1^

Each report has its own specific recommendations. *Advancing the Science* suggests the government support research on human health effects from climate change and notes that health issues are an important way to get people to focus on climate change in general.

*Limiting the Magnitude* recommends the United States set a future limit in the form of an emissions budget on carbon. A carbon budget would provide policymakers with a goal that can be measured, tracked, and that can be used to develop emissions reduction strategies. “If we are going to meet [a goal], we are really going to have to get started,” says report panel chairman Robert Fri, a visiting scholar with the think tank Resources for the Future.^23^

Thomas Wilbanks, group leader of the Global Change and Developing Countries Programs at Oak Ridge National Laboratory and chairman of the *Adapting to the Impacts* report panel, says this report recommends the government and the public employ a risk management mindset when crafting adaptation plans.^23^ “What we need is not only a federal response but a national response,” Wilbanks says. “We need a paradigm shift in which everyone thinks about how to adapt.”

The fourth report, *Informing an Effective Response*, includes a chapter on education and communication needs and strategies. It also examines who is making decisions about climate change response and what those decisionmakers need to make better decisions. “Getting a grip on [climate change] now and beginning to decelerate the consequences, decelerate the contributing factors, is extremely important,” says Peter Raven, president emeritus of the Missouri Botanical Garden and cochairman of the *Informing an Effective Response* panel.—Catherine M. Cooney

## Figures and Tables

**Figure:**
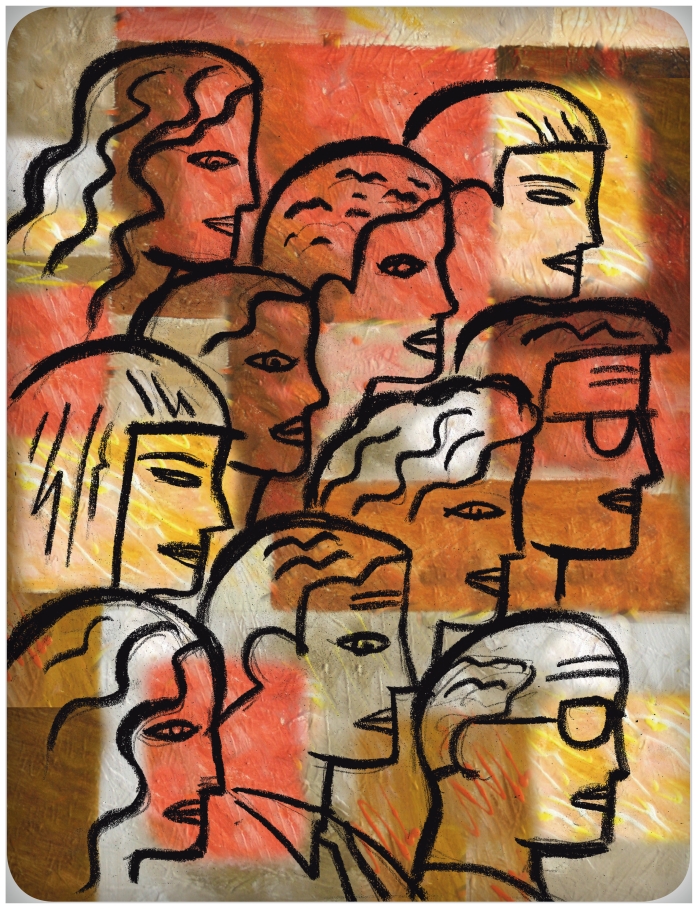


**Figure:**
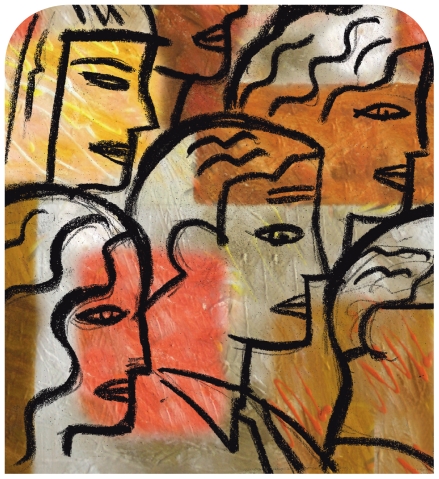
I think we’ve had a major institutional failure to serve and understand the public, then have blamed the public for our failure to communicate effectively. When you think about how big the problems are and how much money we spend on other aspects of them, it is kind of laughable. —Baruch Fischhoff Carnegie Mellon University

**Figure:**
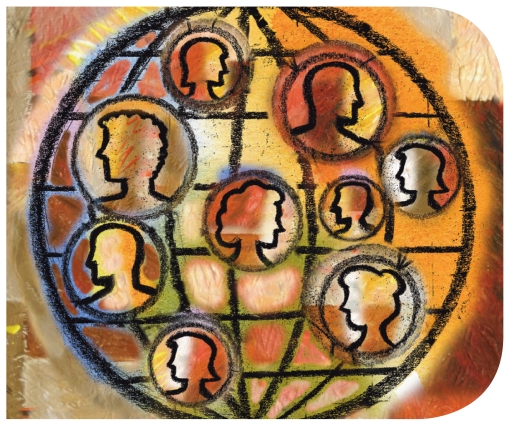
Public health officials have a really important opportunity to explain to people in their jurisdiction . . . that climate change is not just a problem in the future. It is a current problem that will become more pronounced, and our health will suffer. —Edward Maibach George Mason University

**Figure:**